# Effects of maternal toxic substance consumption during breastfeeding on lactic acid bacteria abundance and nutritional content

**DOI:** 10.7150/ijms.87995

**Published:** 2023-09-11

**Authors:** Jesús Alonso Amezcua López, Elisa García Morales, Daniel Pérez-Rulfo Ibarra, Josué Raymundo Solís Pacheco, Blanca Rosa Aguilar Uscanga

**Affiliations:** 1 Universidad de Guadalajara. University Center of Exact Sciences and Engineering. Division of Basic Sciences, Department of Pharmacology. Laboratory of Industrial Microbiology Research. Guadalajara, Jalisco, México.; 2 Universidad de Guadalajara. University Health Sciences Centre. Department of Human Reproduction Clinics, Child Growth and Development, Pediatrics Specialty. Guadalajara, Jalisco, México.; 3 O.P.D. Hospital Civil de Guadalajara "Fray Antonio Alcalde". Division of Pediatrics, Neonatology Service. Guadalajara, Jalisco, México.

**Keywords:** breastfeeding, toxic substances, newborn, microbiota, inhibition

## Abstract

Breast milk is widely recognized as the primary source of nourishment for newborns, making it an unparalleled and indispensable provider of essential nutrients, microbiological components, immunological factors, and energy content. To investigate this further, a cohort comprising 254 breastfeeding women participated in interviews, and milk samples were aseptically collected for subsequent analysis involving bromatological, microbiological, and clinical analysis. The investigation focused on the identification of specific microorganisms in breast milk and their susceptibility to the exposure of toxic substances and controlled medications. Notably, this study places particular emphasis on the significant decline in lactic acid bacteria observed in breast milk when influenced by substances such as cocaine, cannabis, crystal, and morphine. These detrimental agents have been found to adversely affect the growth of microorganisms within breast milk. On the contrary, the outcomes of this study indicate that the utilization of toxic substances does not exert a noteworthy impact on the nutritional quality of breast milk.

## Introduction

Breast milk, is widely recognized as the primary source of nutrition for newborns during their first six months of life, makes it a unique and irreplaceable food as it provides all the necessary nutritional, immunological, microbiological, and energy contents for the optimal development 1. However, specific circumstances may prevent a mother from directly breastfeeding her child 2. It is known that there are very few cases where breastfeeding should be immediately interrupted, as this decision should be the last resort that healthcare professionals should turn to 3. These factors include a mother having an infectious disease that poses a risk to the health of the newborn, such as mothers infected with untreated HIV, hepatitis B or hepatitis C 4, active tuberculosis, advanced-stage cancer, untreated syphilis, and any other disease that can be transmitted through an untreated perinatal infection 5. Currently, the World Health Organization (WHO) recommends continuing breastfeeding for all HIV-positive mothers who are undergoing treatment with Antiretroviral Therapy (ART) 6 and whose viral load is < 50 copies/mL, as it has been demonstrated that the efficiency of ART carries a transmission risk less than 2% compared to when breastfeeding is discontinued 7.

The consumption of toxic substances (illicit drugs and control medications) by the mother jeopardizes exclusive breastfeeding and the health of the newborn, causing a significant global health problem. 8. This makes it necessary to suspend this practice, as these compounds can cause neonatal withdrawal syndrome 9. The consumption of such substances, including nicotine, cocaine (COC), alcohol, amphetamines (AMP), cannabis (THC), phencyclidine, methamphetamines (M-AMP), opioids (OPI) and barbiturates (BAR), which can cross the enteromammary barrier due to their lipid affinity and reach the milk in viable form, causing various systemic alterations in the newborn 10. These alterations include excitability, disorders in the central nervous system, vomiting, muscular paralysis, tachycardia, respiratory failure, and death 11, 12.

One of the most consumed harmful substances during lactation is alcohol. Due to its social consumption and the unknown minimum safe consumption level for breastfeeding, it is recommended not to consume it 13. Regular alcohol consumption can inhibit prolactin, decrease milk production by 10 - 25 %, and block the release of oxytocin 14, 15. This compound can be transferred in small amounts to breast milk, changing its organoleptic characteristics such as taste and smell. Metabolically, newborns are incapable of oxidising alcohol, having negative effects on their behavior, sleep patterns, psychomotor development, and in the worst-case scenario, future kidney problems 16, 17.

Tobacco consumption during breastfeeding is another important factor to consider. Its consumption can change organoleptic properties, causing breast refusal 9. The complications that arise include the inhibition of prolactin release, low milk production, and interference with ejection of breast milk 16. Nicotine, which is the main component of tobacco, can be present in breast milk as cotinine and cause adverse effects such as inadequate weight gains and more frequent colic in the infant 18. Additionally, passive exposure to tobacco smoke increases the risk of sudden infant death syndrome, respiratory infections, cough, and asthma 17. However, due to the significant benefits that breast milk provides to the newborn, it should not be restricted, and alternative pharmacological treatments should be considered to reduce the mother's use of these substances as much as possible 18, 19.

Due to the importance of breastfeeding for the newborn's nutrition, the WHO, United Nations Children's Fund (UNICEF), the American Academy of Pediatrics (AAP), and the Spanish Association of Pediatrics (AEP) recommend evaluating and promoting breastfeeding according to these criteria, as the benefits that breastfeeding provides to the child will be reflected both in the short and long term 12, 20. Breast milk acts as a vehicle for probiotics microorganisms 21, which are responsible for colonizing the newborn's gut, helping to strengthen their immune system and improve their digestive processes by protecting the intestinal lumen 22. For these reasons, it is considered the newborn's first vaccine. Additionally, breast milk provides all the necessary nutritional and energy content, including proteins, lipids, carbohydrates, oligosaccharides, vitamins, and minerals 1, 23.

The breast milk microbiota represents a fundamental factor for newborns, which is why the importance of exclusive breastfeeding for the first 6 months of life and continuing it for at least two years in combination with complementary feeding 23, 24. It is important to mention that in México, there is no established control standard or uniform criteria for values that can be considered satisfactory in unprocessed breast milk. However, according to international standards in the United Kingdom, bacterial growth should not exceed 1 X 10^5^ CFU/mL before pasteurization 25. Similarly, the Human Milk Banking Association of North America (HMBANA) and the European Milk Banking Association (EMBA) have issued a statement warning about the dangers of inappropriate use and consumption of breast milk 26, 27, which should not contain pathogenic bacteria, or no more than 1 X 10^4^ CFU/mL 28.

Breastfeeding should ensure that newborns receive safe and nutritionally optimal feeding according to their needs and requirements. In this study, we investigated how the use of toxic substances, as well as the consumption of alcohol, tobacco, and coffee, affect the nutritional quality and the presence of lactic acid bacteria (LAB) in breast milk.

## Materials and Methods

### Raw material

Breast milk samples were collected based on donations from lactating women after the fifteenth day postpartum (mature milk), who were admitted to the obstetrics service of the Hospital Civil “Fray Antonio Alcalde” (HCFAA) in the city of Guadalajara, México. These samples were collected following the health control criteria of the Department of Health through the Pediatrics Division, Neonatology Service of the HCFAA. These samples were collected from both clinically healthy mothers and those with positive toxicology screening, such as COC, THC, AMP, M-AMP, OPI, and BAR, as well as mother's self-reported consuming alcohol and tobacco during lactation. The standards for toxic substances were selected according to the frequency of consumption and discarding only those patients whose milk production was less than 3 mL per breast, since this amount is insufficient for the analyses.

The study included a total of 254 lactating mothers who were categorized into groups based on their consumption of different substances. These groups including a control group of 151 clinically healthy mothers (evaluated through clinical analysis by the central laboratory of HCFAA). Additionally, specific groups were formed for mothers with positive toxicology screenings with a total of 25 patients, as well as those who consumed alcohol (32 patients), tobacco (29 patients), and coffee (17 patients).

Donor mothers selection for each group was accomplished using convenience non-probabilistic sampling.

Donor mothers were informed about the study and the signing of informed consent and confidentiality letters was carried out in accordance with the provisions of the general health law for health research 29, title 2^nd^, chapter I, article 17, section I, minimal risk research, always taking care of the patient's integrity and considering the ethical criteria established by the research ethics committee of the HCFAA in Guadalajara, México (approval reference number HCG/CEI-0907/22 and research registration 141/22 approved on June 8^th^, 2022), as well as the safeguarding of patients personal information, in accordance with the federal law on protection of personal data of sensitive nature, the protection of sensitive personal data^30^.

### Analysis of the nutritional properties of breast milk

A nutritional content analysis was performed using a LACTOSCAN SA MILK AN-ALYZER® 31 ultrasound equipment adjusted to breast milk samples (LCD display - 4 lines x 16 characters), which analyzes the content of fat, protein, lactose, and total carbohydrates in g/dL.

### Quantification and isolation of lactic acid bacteria present in breast milk from mothers consuming toxic substances

The quantification of LAB in breast milk was carried out using plate counting techniques on selective agar for *Lactobacillus* (agar MRS) 32, grown under anaerobic conditions at 37°C for 24 to 48 hours. Their subsequent identification was done using proteomic tests by the MALDI-TOF mass spectrometry method, which involves the specific and rapid identification of microorganisms by analyzing proteins through a library of a specific mass spectrum for the genus and species, providing results from a culture in less than 24 hours and at a lower cost compared to other molecular biology methods 33.

### Identification of toxic substances presence in breast milk through qualitative analysis

Descriptive surveys, containing both open-ended and closed-ended questions, were conducted with enrolled donor mothers to gather verbal information regarding their use of toxic substances and the frequency of their usage. Subsequently, collected milk samples underwent qualitative analysis for the identification of substances of abuse using the SureStep™ Drug Screen Card I kit from Diagnóstica Internacional 34, which is based on lateral flow immunochromatography and is a one-step in vitro test. For comparison purposes, this kit was evaluated by intentionally contaminating breast milk with the following standards: pentobarbital, fentanyl, midazolam, buprenorphine, morphine, benzodiazepines for controlled medications, and COC, THC, ecstasy, lysergic acid diethylamide (LSD), crystal for toxic substances (the concentrations used are presented in **Table 1**). The standards were obtained through an approved distributor for research in accordance with the General Health Law and Article 479 of the current penal code in México 35, 36. Mothers with positive results in their milk samples were subsequently confirmed with urine toxicology tests.

### In vitro evaluation of the effect of toxic substances and control medications in microorganisms isolated from breast milk

Different microorganisms were identified in breast milk, such as Lacticasibacillus rhamnosus, Limosilactobacillus reuteri, Lactobacillus lactis, Lacticasibacillus casei, Lacticasibacillus paracasei, Levilactobacillus brevis, Bacillus cereus, Bacillus subtidis, Staphylococcus lentus, Staphylococcus haemolyticus, and Enterococcus faecalis. Their resistance capacity to various toxic substances and controlled medications was evaluated on MRS and Mueller-Hinton 37 agar through surface extension culture. Wells containing controlled doses of the substances mentioned in **Figure 5**, relative to the lethal dose in humans, were placed on the agar. Subsequently, they were incubated at 37°C for 24 to 48 hours, and the inhibition zones produced by the different substances were measured.

### Statistical analysis

Differences in the nutritional content of milk samples, microbiological content, and maternal toxic substances use were assessed according to the data distribution with the Shapiro-Wilk test. Parametric data were analyzed using Tukey's one-way ANOVA with Honest Significant Difference (HSD) 38. Non-parametric distributions were evaluated with Kruskal-Wallis one-way ANOVA with Mann-Whitney-Wilcox procedure and post hoc multiple comparisons tests with Bonferroni significance correction 39. Statistical and graphical analyses were performed with R and Statgraphics 19® software 40, 41. As well as Microsoft Excel for database processing 42.

## Results

### Factors that may interfere with the process of exclusive breastfeeding

**Figure 1:**
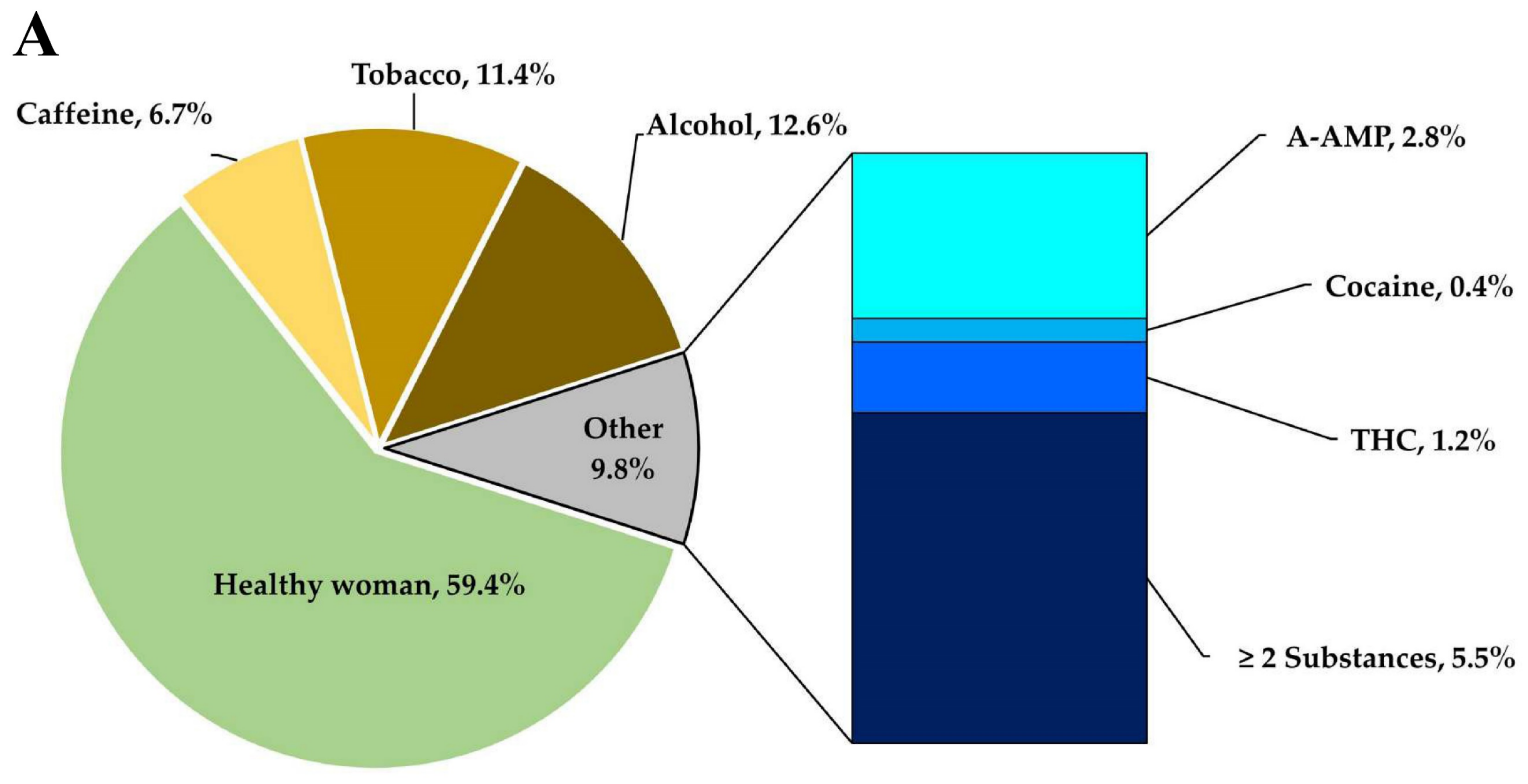

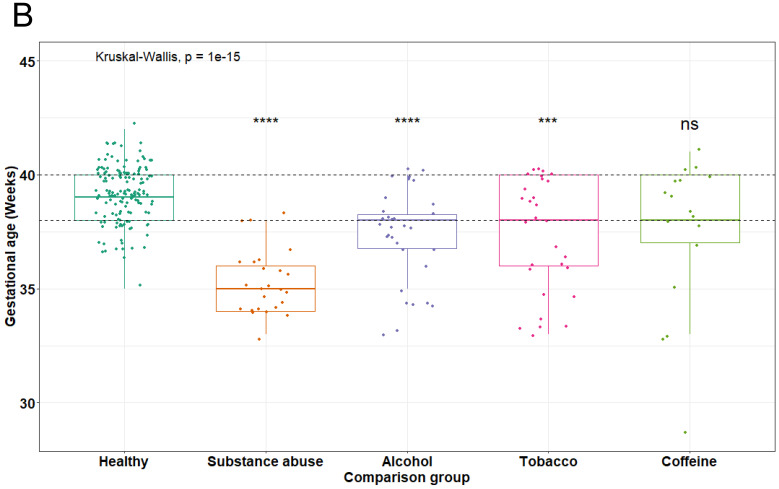
Patterns of substance consumption in relation to gestational age. (A) Shows the frequency of toxic substances use during the lactation period. (B) Depicts the variation in gestational age in relation to the consumption of substances of abuse (**** p < 0.0001; *** p < 0.001; ns, non-significative).

This study considered a total donor mothers of 254 aged 14 to 46 years (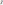
= 26, Mode = 23). The women underwent evaluation tests, including open and conditioned questionnaires, clinical analyses, toxicological tests, and nutritional analyses, to be classified into one of five groups using a non-probabilistic convenience model. The groups included a control group of healthy donor mothers, which was compared to groups of donor mothers who consumed toxic substances and had positive toxicological tests for COC, AMP, M-AMP, OPI and THC. As well as groups consumer's alcohol, tobacco, and coffee, all during the lactation period. These categories correspond to the factors that compromises a successful lactation, as shown in **Figure 1A**, which displays the frequency with which these substances were used by the mothers.

In the others' section of **Figure 1A**, which corresponds to 9.8 % of toxic substances cases, it was found that 2.8 % of mothers were ingesting methamphetamines, 0.4 % were inhaling cocaine, and 1.2 % were smoking marijuana. It is worth noting that around 5.5 % of mothers were using two or three types of substances used simultaneously.

Another important data point related to toxic substances use is the gestational age of the newborns, which can be observed in **Figure 1B**. The figure depicts a clear relationship between toxic substances during pregnancy and a reduction in gestational age. Typically, pregnancies for healthy mothers last between 38 to 40 weeks, with a mean of 39.17 ± 1.20 weeks in this cohort. However, donor mothers who consume toxic substances have a significant reduction in gestational age, with a mean of 35.8 ± 1.4 weeks (p < 0.0001). This decrease can lead to an optimal gestational age reduction of up to one month and an increased risk of premature birth.

Regarding the groups of mothers consuming alcohol (p = < 0.0001) and tobacco (p = < 0.001), there is a significant difference (p = < 0.0001) in gestational age compared to healthy mothers. The statistical analysis demonstrated no significant (ns) difference in gestational age between mothers consuming caffeine and healthy mothers.

**Figure 2:**
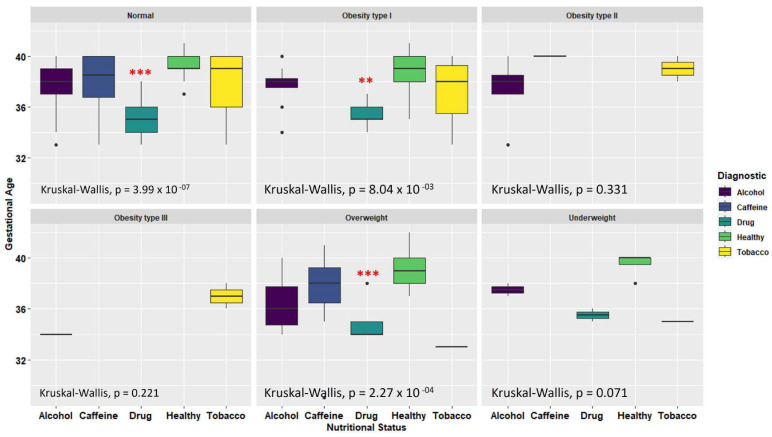
Relationship of maternal nutritional status and toxic substance consumption with the reduction of gestational age (Kruskal-Wallis test, ** p = 0.01, *** p = 0.001).

To compare the results regarding the reduction in gestational age among mothers who were consuming a toxic substance and to determine if this reduction was not attributable to the mothers low weight or nutritional status. A statistical analysis was conducted using the nutritional status according to the Body Mass Index (BMI). A Kruskal-Wallis test resulted in a non-significant contribution to the double factor analysis (p = 0.2072). Notably, regardless of the nutritional status in which the mothers were situated, the consumption of any toxic substance led to a decrease in gestational age, as observable in **Figure 2**. These findings suggest that the reduction in gestational age among the donor mothers is not related to the nutritional status, but rather to the consumption of toxic substances.

### Effect of toxic substances consumption on the nutritional properties of breast milk

**Figure 3:**
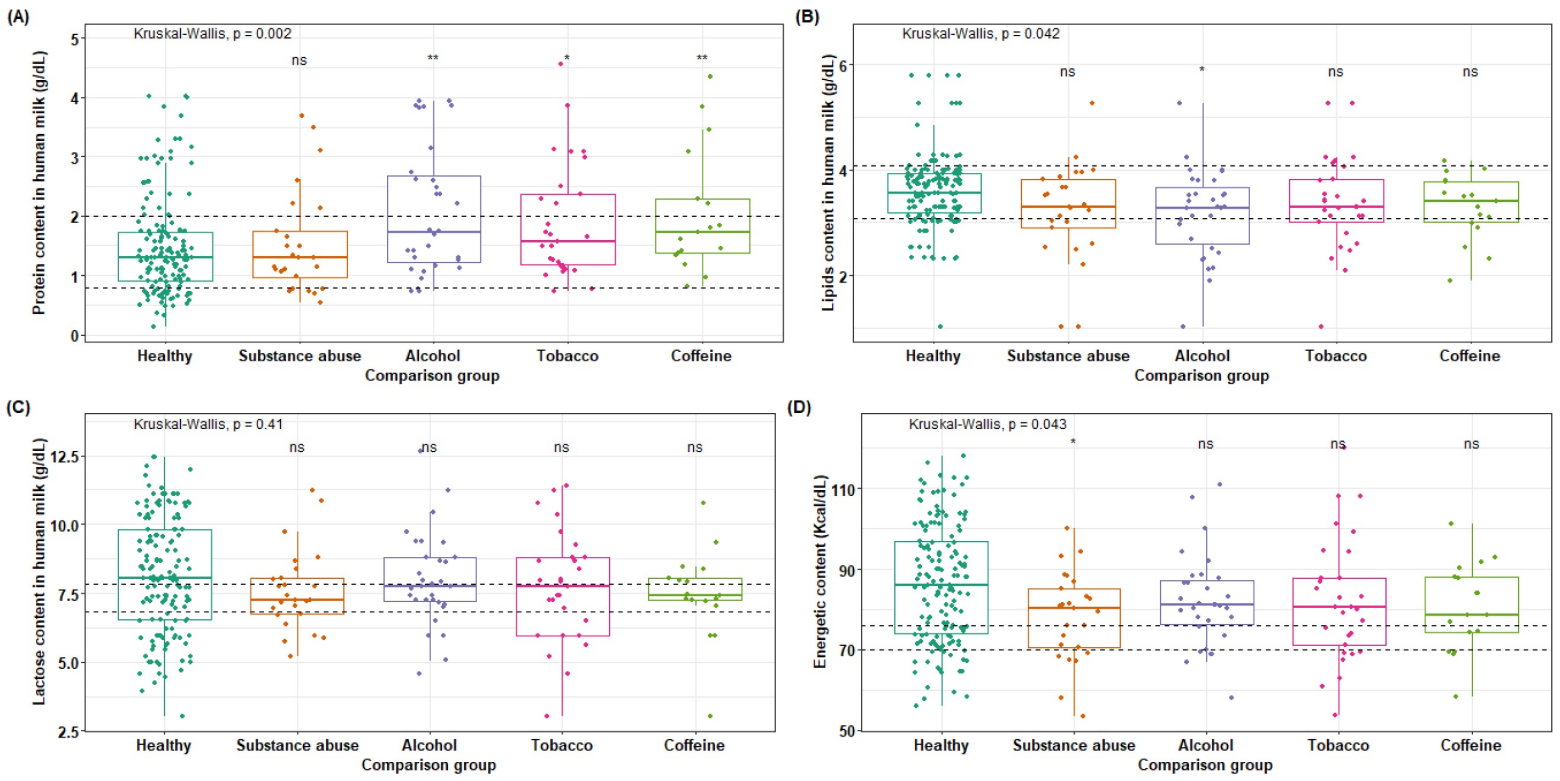
Macronutrients available in breast milk of mothers who use toxic substances. (A) The protein content present in breast milk was compared between the groups of mothers who use toxic substances, alcohol, tobacco, and caffeine, against the values of healthy mothers (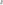 = 1.48 g/dL); (B) total lipid content in breast milk of the five groups, 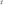 = 3.60 g/dL for healthy mothers; (C) lactose content, 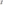 = 8.16 g/dL for healthy mothers; (D) energy content, 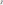 = 86.30 Kcal/dL for healthy mothers.

This study investigated variations in the nutrient composition of breast milk in relation to the use of toxic substances in donors during lactation. Donors were divided into groups as described in section (Raw material). Analysis of the protein content of breast milk (**Figure 3A**) revealed a standard of 1.39 ± 0.6 g/dL, with no significant differences found between the control group (clinically healthy) and the group of individuals who consume toxic substances (p = 0.002). However, a difference was observed between healthy mothers and alcohol users, although it was not statistically significant. Total lipids in breast milk (**Figure 3B**) had a standard of 3.58 ± 0.5 g/dL, with no significant differences found between groups (p = 0.042).

Regarding lactose values (**Figure 3C**), a slight increase in lactose content is observed in healthy mothers compared to the four groups within the standard range of 7.34 ± 0.5 g/dL, although no significant difference was reached (p = 0.41). However, the alcohol and tobacco groups have means that fall within the upper limit of the standard value. In (**Figure 3D**), the energy content in Kcal per 100 mL is shown, where all groups exceed the maximum standard limit of 70-76 Kcal/dL. However, a slight significant difference is only observed between the groups of donors who are consumers of toxic substances and clinical healthy donors, while no significant difference is found with the other groups (p = 0.043).

The amounts of saturated and unsaturated fats were also evaluated, but these did not show statistically significant variations. This indicates, that although there are variations in the nutrients of the milk, the impact is minimal and does not compromise the nutritional quality of the milk for proper feeding. Nevertheless, doctors should evaluate this situation regarding the risk of toxic substances ingestion to the newborn through breast milk.

### Identification of traces of toxic substances in breast milk

After collecting milk samples from donor mothers who are consumers of toxic substances, confirmed through toxicological tests in urine, the milk was evaluated for the presence of these substances. To do this, corresponding control samples were first performed with the concentrations of substances shown in **Table 1**, in order to evaluate the efficiency of the kit that determines the presence of COC, THC, OPI, AMP, and M-AMP. The mentioned concentrations were calculated according to the information provided by the international immunodiagnostics and the Substance Abuse Mental Health Services Administration (SAMHSA) of the USA. Controlled drugs and toxic substances were handled according to the provisions of Chapter VII, Article 479 of the General Health Law, in its latest reform of May 16^th^, 2022, regarding the maximum allowable dose guidance 29, 43, 44.

Once the sensitivity of the toxic substances test kits was validated in both intentionally contaminated urine samples and breast milk samples, analyses were performed on milk samples from donor who are consumers of toxic substances. Out of the 254 milk samples collected, it was identified that 9.8% of the samples tested positive for toxic substances, as shown in **Figure 1**, while all samples tested negative for amphetamines and opioids.

### Quantification of microorganisms in breast milk of toxic substances consumers

**Figure 5:**
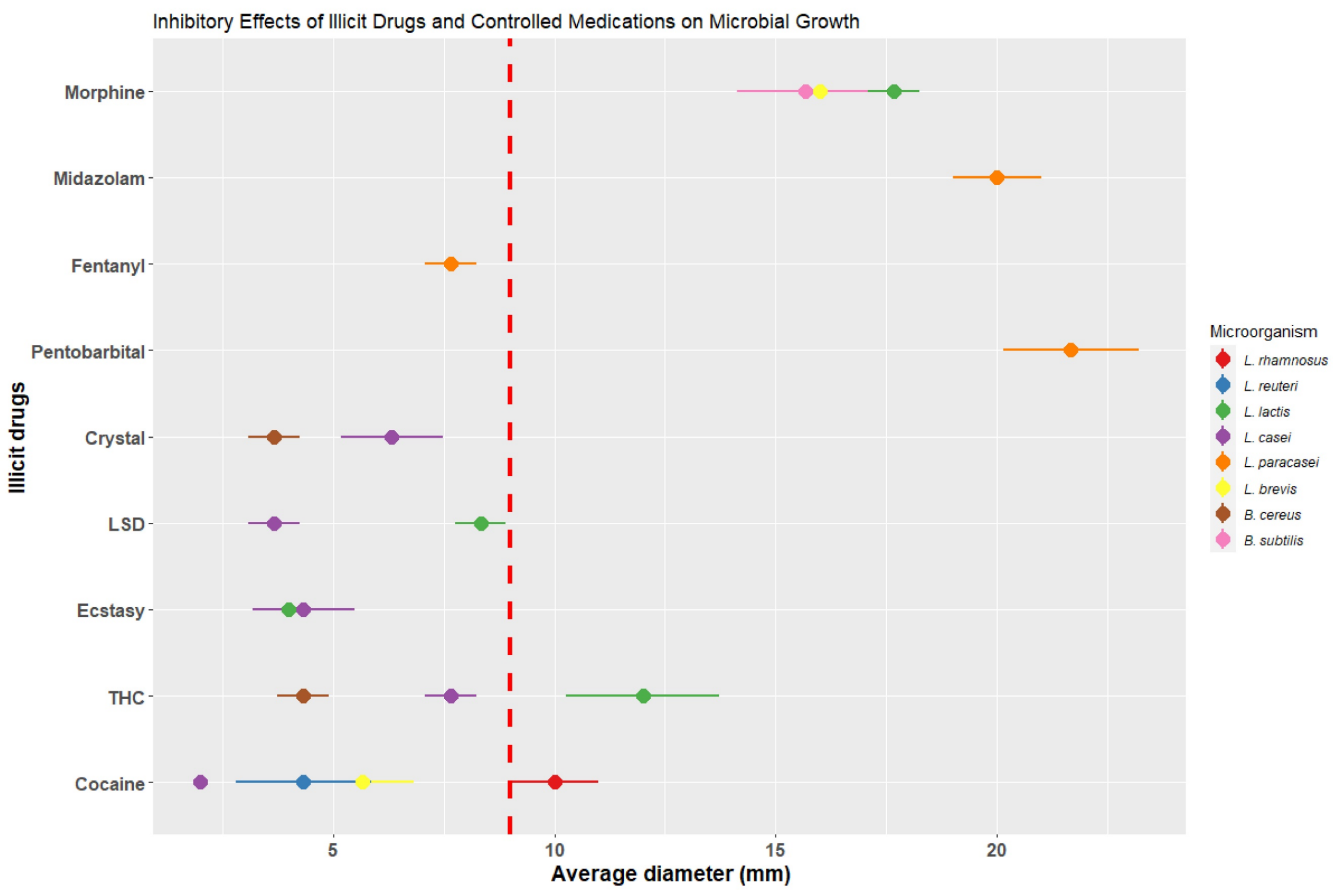
Inhibitory effects of toxic substances and controlled medications on microbial growth (The red dashed line represents the average value of the antibiotic control used for the inhibitions, which measures 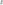 = 9 ± 1.56 mm in diameter).

Once the milk samples were collected from mothers consuming different harmful substances, the cultivation and quantification of LAB on MRS agar was carried out for each group previously mentioned. **Figure 5** shows the variations in CFU/mL, where it can be observed that the group of donors who are consumers of toxic substances is the most affected. While established parameters for normal mothers range from 1 x 10^4^ to 1 x 10^6^ CFU/mL, for this group of consumers of toxic substances, the mean is 9.59 x 10^3^ CFU/mL, indicating a decrease of one logarithm relative to the minimum limit. The statistical analysis using Kruskal-Wallis test, demonstrating a significant difference in the decrease of LAB with respect to healthy mothers (p < 0.001).

In mothers who habitually consumed alcohol (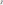 = 1.29 x 10^6^), tobacco (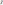 = 1.53 x 10^6^) and coffee (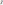 = 1.45 x 10^6^), no significant decrease was observed. This is because these groups exhibited a normal distribution within normal ranges.

### Inhibition of microbial growth in breast milk upon exposure to toxic substances and controlled medications

To assess the effect of toxic substances and controlled medications on microbial growth in breast milk, diverse microorganisms were isolated and identified from samples of milk from healthy mothers. This included BAL, saprophytic, and some pathogenic bacteria. Subsequently, the microorganisms were exposed to specific concentrations of substances relative to the lethal dose in humans per kilogram of body weight: pentobarbital 0.28 mg 46, fentanyl 0.04 mg 47, midazolam 0.10 mg 48, buprenorphine 0.32 mg 49, morphine 0.60 mg 50, COC 1.20 mg 51, THC 10 mg 52, ecstasy 5 mg 53, LSD 0.60 mg 54, and crystal 0.20 mg 55, as shown in **Figure 5**.

Once each of the microorganisms was incubated with the corresponding exposure to each substance and validated in triplicate, it was observed that 58.33 % of the microorganisms were affected by some of the toxic substances and 41.66 % by some of the controlled drugs, preventing their microbial growth in the presence of the substances. As for lactic acid bacteria such as *L. rhamnosus* and *L. reuteri*, their growth was affected in the presence of COC, crystal, and THC, resulting in inhibitions ranging from 3.67 ± 0.58 to 5.67 ± 1.15 mm in diameter. However, the inhibition caused by toxic substances was lower compared to the control (using a degradation-resistant antibiotic such as carbencillin at a concentration of 0.1 mg/disk 56), which presented an average diameter of 9 ± 1.56 mm. The species *L. brevis* was inhibited when exposed to all 5 types of toxic substances, being most affected by crystal with a diameter of 6.33 ± 1.15 mm and THC with a diameter of 7.67 ± 0.58 mm.

Regarding the controlled medications, *L. reuteri* exhibited an inhibition of 16 ± 1 mm in diameter, surpassing the average value of the control. *L. casei* was most affected by midazolam (20 ± 1 mm), fentanyl (7.67 ± 0.58), and pentobarbital (21.67 ± 1.53 mm), exceeding the control with carbencillin (**Figure 5**). It is important to highlight that the microorganisms *L. lactis, L. casei*, and *S. haemolyticus* did not experience inhibition in their growth in the presence of toxic substances and controlled medications, which indicates a resistance to such substances.

These bacterial inhibition assays allow us to observe the direct effect of toxic substances and controlled drugs on the cultivable microbiota of healthy mothers' milk. As a result of these analyses, a decrease in the number of bacteria was observed, which was reflected in the plate emptying counts.

When comparing these results with the quantification of cultivable microorganisms for LAB, counts below the established minimum limits of up to 2 Log were observed in milk samples from consuming donor mothers of toxic substances, with values between 1 x 10^3^ and 1 x 10^4^ CFU/mL, with an average of 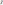 = 9.59 x 10^3^ ± 3.21 x 10^2^ CFU/mL, compared to a standard value of 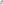 = 1.4 x 10^6^ ± 1.0 x 10^6^ CFU/mL for healthy mothers.

## Discussion

### Relationship between toxic substance and gestational age

After analyzing the data regarding the 5 groups and interpreting the statistical analyses with respect to toxic substances use and the gestation weeks period, it was identified that mothers who mainly consumed cocaine, methamphetamines, and marijuana had a lower number of gestational weeks (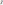 = 35.8) compared to healthy mothers, demonstrating a significant difference between the two groups. The National Institute on Drug Abuse of the United States that drug use during pregnancy and lactation increases the risk of spontaneous abortions, as well as causing migraines, seizures, and high blood pressure that can directly affect the fetus 43. In addition, smoking marijuana or tobacco during the gestational period increases the risk of stillbirth by 1.8 to 2.8 times 57, 58.

### Evaluation of the quality and safety of breast milk from toxic substances consuming donors

In the analysis of the nutritional content of milk from mothers who consume toxic substances, it is observed that although there is a slight variation in nutrient levels, such as protein, between mothers who consume alcohol and those who do not, these differences are not statistically significant (p = 1.1 e^-12^) and would not significantly impact infant nutrition.

However, the real risk for the infant lies in the concentration of toxic substances that may be present in the milk and thus enter their organism. Our analyses identified traces of drugs in some milk samples, as mentioned by Lawrence et al. in 2016, that the consumption of toxic substances presents a high risk for the newborns, since most drugs can reach the alveolar cells of the breasts in free form and are available in the milk. The drugs with the highest receptors in the breasts are phenytoin, salicylate, and diazepam 59.

Philip O. Anderson also mentions in his article published in June 2022 that in patients with diseases during lactation such as arthritis that require pharmacological treatment with controlled medications such as opioids, they should be evaluated and considered as the last line to use them 60. Anderson suggests that although the concentrations of these controlled medications in the milk are lower than those used for neonatal analgesia, alternative medications should be chosen. In 2012, Saguer reported a case of cocaine poisoning in a three-month-old baby in Colombia, who was admitted to the emergency unit due to seizures caused by cocaine 61. Furthermore, studies conducted by Pediatrics in 2018 revealed the presence of cannabis metabolites in breast milk, confirming the transmission of various toxic substances through breastfeeding 20.

### Alcohol, tobacco, and their relationship with breast milk

Regarding the results obtained from breast milk analyzed from mothers who consume alcohol and tobacco, we observed that the nutritional values with respect to the total content of lipids and carbohydrates remain unchanged, except for the protein content where a slight increase is observed, but it remains not significant. Authors such as Acosta in 2020 observed that mothers who consume alcohol and tobacco had premature abandonment of breastfeeding, on average only breastfeeding for two months 62. The American Academy of Pediatrics notes in its latest update in July 2020 that alcohol consumption does not have a safe dose during breastfeeding and decreases milk production, posing a potential risk to the infant's health 63.

Rowe et al. in 2013 reported data from a study titled "The transfer of alcohol to breast milk," which evaluated 12 women who ingested 0.3 g/kg of ethanol and found that the mean maximum concentration of ethanol in milk was 320 mg/L evaluated in the 4 hours after ingestion 46. The study also found that the ingestion of 1.5 g/kg significantly reduced milk production and that constant consumption of 21 drinks daily caused psychomotor delay in infants 65.

Regarding the concentration of nicotine and cotinine with an average consumption of 17 cigarettes per day, it was observed that the concentrations in milk decreased by 50 % to 66 %, equivalent to 25.2 mg/kg/d present in breast milk 64, 65. Our study identified that out of a total of 254 women, 30, equivalent to 11.4 % of the total, smoked an average of one pack of cigarettes per day (20 cigarettes), which increases the concentration of nicotine in the breast milk they produce, as reported by Rowe 64, 66, 67.

The physicochemical results for these groups, for both proteins, lipids, and carbohydrates, although showing slight variations, were statistically non-significant, and the nutrients were within the established ranges for healthy women. In November 2022, Philip O. Anderson discussed alcohol consumption disorder during lactation as a primarily psychological issue for both mother and child's health. As a result, treatment with medications such as naltrexone, baclofen, gabapentin, ondansetron, and topiramate is recommended as they are unlikely to harm the infant 68.

### Effects of toxic substances on microorganisms isolated from breast milk

Research conducted by López M. in 2017 and Amezcua in 2018 demonstrated that mothers who were under the influence of toxic substances showed a microbiological count by plate pouring on MRS agar of 1 x 10^4^ or 1 x 10^5^ in normal mothers 69, 70, whereas in mothers who were under the influence of toxic substances, the counts decreased significantly to 1 x 10^2^ 71. When we subjected the microorganisms isolated from breast milk to growth in the presence of different toxic substances, it was shown that some microorganisms did indeed die, or their growth was inhibited either by exposure to these compounds.

In 2012, Arroyo et al. from the National Institute of Perinatology Isidro Espinosa de los Reyes conducted a study on 57 lactating mothers who were consumers of toxic substances such as COC, THC, OPI, AMP, and benzodiazepines 72. The study demonstrated the presence of these substances in breast milk samples, confirming the feasibility of drug transmission in milk. In 2020, the Organization of Teratology Information Specialists mentioned that cocaine can pass directly into milk in any of its forms, posing a serious risk to the child's health, causing irritability, seizures, and even death 73. The Fourth Trimester Project at the University of North Carolina in 2022 states that the consumption of marijuana during lactation causes the main component of this THC, to be stored in body fat and remain there for up to 30 days, thus demonstrating that the milk of mothers who consume these toxic substances contains high concentrations of THC 75. The information provided by these authors and research institutions reinforces the results obtained by our research group regarding the positivity of toxicological screenings in breast milk.

Regarding the relationship between the presence of these drugs of abuse and their relationship with microbial growth Ramos (2012) demonstrated that the leaf extract of *Erythroxylum coca* has an inhibitory effect on the growth of the bacteria ATCC *Porphyromonas gingivalis* as this leaf contains alkaloids related to cocaine 76. In 2019, a study related to the effect of the cannabinoid system demonstrated that acute stress in mice, which were administered certain concentrations of this substance, increased the expression of the intestinal endocannabinoid system degradation enzyme 77.

With this information provided by these authors in in vitro experiments with different types of substances, demonstrates the direct relationship shown by our research group on the decrease of LAB in breast milk samples from mothers who consumed these substances. Once certain microorganisms were isolated from the milk and exposed to concentrations of and controlled medications in relation to the lethal dose in humans per Kg of body weight, it was observed that *L. reuteri, L. casei,* and *L. rhamnosus* were affected in their growth. These microorganisms were exposed to cocaine, methamphetamine, marijuana, morphine, pentobarbital, fentanyl, and midazolam, and their growth was found to be negatively impacted.

However, this inhibition not only occurred in LAB, but also, in other groups of microorganisms, including pathogenic and saprophytic bacteria, in which controlled drugs, LSD, ecstasy, and cocaine were the substances that generated the most inhibition. Thus, suggesting that the constant use and consumption of toxic substances and controlled medications during lactation could have a detrimental effect on the proper growth of microbiota naturally present in milk. Within our study, the toxic substances most commonly used by donor in breastfeeding stage were crystal (M-AMP) in first place, followed by THC and COC. Most of them not only consumed a single substance but even had mixtures of these three toxic substances at the same time, and to a lesser extent, they consumed ecstasy, LSD and fentanyl.

## Conclusions

Result of study states the importance of monitoring lactating mothers who use toxic substances, as they often deny their consumption during prenatal care and lactation, putting the newborn health at risk. In studies conducted on breast milk samples from donor mothers consuming toxic substances, qualitative analysis identified the presence of cocaine, methamphetamines (primarily crystal meth), and marijuana. However, this analysis was limited to observing the presence or absence of the toxic substances. It has been shown that although the consumption of these substances does not have a significant effect on the nutritional content of breast milk, it does present a potential risk to the development of the milk's own microbiota. It mainly affects the growth of acid lactobacilli when substances such as morphine, midazolam, and pentobarbital are used, which have a greater bacterial inhibition compared to the control with antibiotics. In addition, the use of toxic substances such as crystal meth, THC, and COC affects the growth of *Lactobacillus* and is more frequently consumed in our population.

With the results obtained, it highlights a social problem that affects the health of newborns breastfed with milk from donor mothers consuming toxic substances. For these reasons, it is necessary to implement preventive measures and support in hospitals during pregnancy and lactation, as well as offer pharmacological alternatives to donors with addiction issues, avoiding breastfeeding for their children until they can safely reintegrate. Otherwise, this habit can have negative consequences for the newborn health, inadequate microbial colonization in the intestine, and generate complications in adulthood. These advancements aim to ensure that healthcare personnel identify the risks that arise when a lactating mother experiences addiction problems, enabling immediate action to guarantee health and improve the quality of life for future generations.

## Figures and Tables

**Figure 4:**
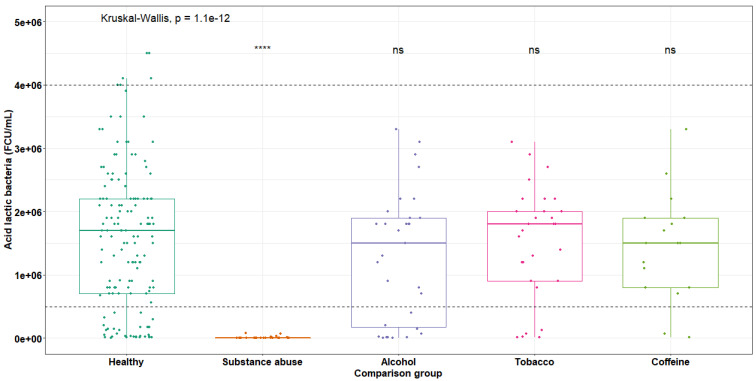
Variations in LAB content in breast milk of toxic substances consuming mothers (maximum standard range 4.0 x 10^6^, minimum 5.0 x 10^5^ CFU/mL), average for healthy donors 1.64 x 10^6^ CFU/mL

**Table 1:** Concentration of controlled drugs and toxic substances for the validation of the Surestep Drug Screen Card I Kit.

Toxic substances	Concentration (µg/ 10 ml)	Urine test (POS/NEG)	Breastmilk test (POS/NEG)
Control	0	NEG	NEG
**Controlled drugs**			
Pentobarbital (BAR)	3	POS	POS
Fentanyl (OPI)	3	POS	POS
Midazolam (OPI)	3	POS	POS
Buprenorphine (BZD)	0.1	POS	POS
Morphine (OPI)	3	POS	POS
Buenzodiacephine (BZD)	3	POS	POS
**Toxic substances**			
Cocaine (COC)	25	POS	POS
Marihuana (THC)	15	POS	POS
Ecstasy (M-AMP)	10	POS	POS
Lysergid (LSD)	10	POS	POS
Crystal (M-AMP)	10	POS	POS

The abbreviation **POS** in rows three and four signifies that the qualitative test yielded a **POSITIVE** result for that concentration of the toxic substances or controlled drug. The abbreviation **NEG** in rows three and four signifies that the qualitative test yielded a **NEGATIVE** result for that concentration of the toxic substances or controlled drug. The concentrations used for the substances were determined according to the minimum detectable values of the kit used 45.

## Abbreviations

AAP: American Academy of Pediatrics
AEP: Asociación Española de Pediatria
AMP: amphetamines
BAR: barbiturates
CFU: colony forming units
COC: cocaine
d/dL: grams per deciliter
d: day
EMBA: European Milk Banking Association
g: grams
HCFAA: Hospital Civil "Fray Antoni Alcalde"
HMBANA: Human Milk Banking Association of North America
HSD: Honest Significant Difference
Kg: kilograms
LAB: lactic acid bacteria
LSD: lysergic acid diethylamide
M-AMP: methamphetamines
mL: milliliters
NEG: negative
OPI: opioids
POS: positive
SAMHSA: Substance Abuse Mental Health Services Administration
THC: cannabis
UNICEF: United Nations Children's Fund
WHO: World Health Organization
arithmetic mean
